# Fast and slow lanes of the vagus

**DOI:** 10.1113/EP090868

**Published:** 2024-10-18

**Authors:** Song T. Yao, Julian F. R. Paton

**Affiliations:** ^1^ Department of Anatomy and Physiology The University of Melbourne Parkville Victoria Australia; ^2^ Manaaki Manawa – The Centre for Heart Research, Department of Physiology, Faculty of Medical & Health Sciences University of Auckland Grafton Auckland New Zealand

This issue of *Experimental Physiology* contains symposium reports that were presented 5 September at the Congress of the International Society of Autonomic Neuroscience—ISAN 2022 in Cairns, Australia.

Physiologists are highly skilled at measuring fast, dynamic bodily responses in real time, such as the dramatic reduction in heart rate when the cardiac vagus nerves are activated. However, they are less adept at measuring responses that manifest over hours, days or even weeks. This symposium brought together leading experts in the field to discuss aspects of the fast and slow functions of the cardiac vagus efferent outflow. Using cutting‐edge technology, such as three‐dimensional single‐cell RNA sequencing of the intrinsic cardiac ganglia, in vivo somatic gene transfer, optogenetics and whole‐cell patch clamping, this symposium reported and discussed substantial new knowledge about both the fast and slow functions of the cardiac vagus. It considered how the structure and function of the cardiac vagus divide and conquer to ensure optimal functioning, in addition to protection in disease states. Multiple cardiac functions were considered, such as chrono‐, iono‐ and dromotropism and coronary blood flow at rest and during exercise. It revealed the novel integrative crosstalk between the cardiac vagus and other organs, such as the gastrointestinal tract, in health and disease.

Gee et al. (2024) describe an integrative framework based on computational modelling to combine disparate and multiscale data on the two vagal control lanes of the cardiovascular system. New molecular‐scale data, perhaps most importantly single‐cell transcriptomics, have dramatically increased our understanding of the neuronal states underlying vagally mediated fast and slow regulation of cardiac physiology. Computational models at the cellular scale, built from these datasets combined with circuitry, neuronal and organ‐scale physiology data, allow the creation of multisystem, multiscale models that enable in silico exploration of fast versus slow lane vagal stimulation. These computational models allow experimental questions on the mechanisms regulating vagal control of the heart to be explored, ultimately improving cardiovascular health.

Booth et al. (2024) addressed the potential of using vagus nerve stimulation as a treatment for heart failure. Heart failure patients often have a poor quality of life, experiencing dyspnoea, fatigue, oedema and depression, and are at high risk of hospital admission and increased rates of mortality and morbidity. Vagal nerve stimulation has been trialed using whole‐nerve electrical stimulation, but the results have been mixed. Why? This might, at least in part, be attributable to the inability to recruit the right vagal fibres selectively and the lack of knowledge about which specific fibres of the fast and slow lanes to target. This review discusses the different populations of cardiac‐projecting efferent vagal fibres, with cell bodies located in the dorsal motor nucleus of the vagus nerve, and describes new methods of selectively targeting these projections as new treatments for heart failure.

Shanks et al. (2024) focus on understanding the role of autonomic non‐adrenergic, non‐cholinergic cotransmitters in the regulation of coronary blood flow. The coronary circulation is essential for maintaining myocardial function. An increase in the metabolic demand of the heart is accompanied by parallel increases in blood flow through the coronary arteries in an adaptive manner via mechanisms including local metabolic factors, mechanical tissue forces, circulating hormonal factors and neural control. However, neural control has focused predominantly on the classical neurotransmitters noradrenaline and acetylcholine. Here, the authors discuss the importance of ‘non‐classical’ neurotransmitters, such as vasoactive intestinal peptide, and how a greater understanding of these might lead to the development of new targeted therapies.

Finally, Ragozzino et al. (2024) present findings on circadian regulation of autonomic tone. Although this has been described in the past, the underlying mechanisms mediating these changes in autonomic neurocircuitry have, so far, been largely unexplored. The dorsal vagal complex, consisting of the nucleus of the solitary tract, dorsal motor nucleus of the vagus and area postrema, are key candidates for the rhythmic control of the autonomic nervous system. Here, Ragozzino and colleagues report that vagal afferent neurons express rhythmic clock genes and demonstrate rhythmic action potential firing and rhythmic spontaneous glutamate release. This short review provides new insights into the neurophysiological principles that dictate nucleus of the solitary tract synaptic transmission and the impact that circadian rhythms have throughout the day.

In summary, this symposium provided an excellent introduction to the fast and slow lane functions of the cardiac vagus nerve. These reviews are timely because they highlight the many breakthroughs in this area, including a greater understanding of the short‐ and long‐term functions of the vagus nerve. The symposium demonstrated how manipulation of the vagus nerve can produce therapeutic benefits in numerous cardiovascular diseases. This complements the ongoing Research Evaluating Vagal Excitation and Anatomical Linkages (REVEAL) trial aiming to understand the therapeutic benefit of vagus nerve stimulation in humans.

## AUTHOR CONTRIBUTIONS

Both authors have approved the final version of the manuscript and agree to be accountable for all aspects of the work. Both persons designated as authors qualify for authorship, and all those who qualify for authorship are listed.

## CONFLICT OF INTEREST

None declared.

## FUNDING INFORMATION

DHAC | National Health and Medical Research Council (NHMRC): Song T Yao, GNT1187962.

## References

[eph13651-bib-0001] Booth, L. C. , Saseetharan, B. , May, C. N. , & Yao, S. T (2024). Selective efferent vagal stimulation in heart failure. Experimental Physiology, 109(12), 2001–2005. 10.1113/EP090866 PMC11607613 37755233

[eph13651-bib-0002] Gee, M. M. , Hornung, E. , Gupta, S. , Newton, A. J. H. , Cheng, Z. J. , Lytton, W. W. , Lenhoff, A. M. , Schwaber, J. S. , & Vadigepalli, R. (2024). Unpacking the multimodal, multi‐scale data of the fast and slow lanes of the cardiac vagus through computational modelling. Experimental Physiology, 109(12), 1994–2000. 10.1113/EP090865 PMC10613580 37120805

[eph13651-bib-0003] Ragozzino, F. J. , Karatsoreos, I. N. , & Peters, J. H. (2024). Principles of synaptic encoding of brainstem circadian rhythms. Experimental Physiology, 109(12), 2006–2010. 10.1113/EP090867 PMC11607608 38308846

[eph13651-bib-0004] Shanks, J. , Thomson, S. , & Ramchandra, R. (2024). Neural control of coronary artery blood flow by non‐adrenergic and non‐cholinergic mechanisms. Experimental Physiology, 109(12), 2011–2016. 10.1113/EP090917 PMC11607609 37029787

